# Rapid Molecular Diagnostics of Tuberculosis: What Do We Have, What Do We Need?

**DOI:** 10.1155/pm/9947140

**Published:** 2026-09-26

**Authors:** Bruno Luukinen

**Affiliations:** ^1^ Department of Clinical Microbiology, Fimlab Laboratoriot, Tampere, Finland

## Abstract

Smear microscopy and culture are conventional methods of tuberculosis (TB) diagnostics. Microscopy is fast and inexpensive but has low sensitivity, whereas culture is highly sensitive but may take several weeks. Molecular diagnostic (MDx) methods were widely introduced into TB diagnostics much later, in the early 2010s, when the GeneXpert system was launched. MDx methods combine the benefits of the conventional methods. Furthermore, significant developments have occurred in recent years. The current selection of available rapid MDx methods covers the full range of TB diagnostics: detection of pulmonary and extrapulmonary TB, narrow‐ to broad‐range rapid drug susceptibility testing, mycobacterial species identification, and *Mycobacterium tuberculosis* (MTB) strain typing. Several issues—often focusing on high assay costs—have still prevented the complete roll‐out of MDx methods over conventional methods in both low‐resource, high‐incidence settings and high‐resource, low‐incidence settings. This review covers topics related to molecular detection of MTB and MTB drug resistance directly from clinical specimens. The aim of the review is to describe currently available methods, how they perform, and what key issues must be addressed for more optimal implementation. Overall, MDx methods currently represent an essential but often accessory tool in the detection of TB and MTB drug resistance. At present, several limitations—including diagnostic equivalence with the reference method, breadth of drug resistance testing, treatment monitoring, and accessibility due to analysis costs and assay complexity—still hinder efficient replacement of the conventional smear microscopy and culture methods. However, new advances in testing methods and strategies are continuously being made.

## 1. Introduction

Laboratory diagnostics of tuberculosis (TB) disease traditionally involve smear microscopy and culture. Despite many recent advances, these methods still hold an important place in bacteriological confirmation of TB. However, neither method is specific for *Mycobacterium tuberculosis* (MTB), the causative agent of TB, but instead may also detect other mycobacteria and aerobic actinomycetes such as those of the genera *Nocardia*, *Rhodococcus*, *Tsukamurella*, *Gordonia*, and *Dietzia* [1]. In many low‐resource settings, anti‐TB drug treatment decisions may be based solely on positive sputum smear microscopy [2]. Without differential diagnosis, this practice may lead—and has led—to a significant proportion of nontuberculous mycobacterial (NTM) infections being misdiagnosed as drug‐resistant TB [2, 3]. In two geographically distinct studies, more than 10% of suspected TB patients in China and 30% of suspected MDR‐TB (multidrug‐resistant TB) patients in Iran were positive for NTM [3, 4]. These findings highlight the need for species‐specific approaches for detection of TB, such as rapid molecular diagnostic (MDx) tests.

Although the first reports of molecular detection of TB date back more than 30 years, molecular TB diagnostics became widely available much later, following the launch of Cepheid′s GeneXpert platform (Sunnyvale, California, United States) in 2009 [5–7]. Since 2010, the World Health Organization (WHO) has recommended the use of MDxs for detection of TB—a list that has expanded considerably since endorsement of the Xpert MTB/RIF assay (RIF [rifampicin]) (Table 1) [8, 11]. Today, rapid molecular testing is an established part of bacteriological confirmation methods of TB. Despite major technical advances from labor‐intensive manual PCR methods to rapid fully automated systems, more affordable and accessible applications are still needed to improve testing coverage. In 2024, only 54% of new TB cases globally were tested with rapid diagnostics at the time of diagnosis [12].

**Table 1:** List and description of WHO‐recommended rapid molecular diagnostic assays for detection of tuberculosis and MTB drug resistance [8–10].

**Initial TB testing**


*Low-complexity automated NAATs*
Assay(s)
Xpert MTB/RIF (Cepheid)
Xpert MTB/RIF Ultra (Cepheid)
Truenat MTB Plus and Truenat MTB‐Rif Dx (Molbio Diagnostics)
(Truenat MTB Ultima [Molbio Diagnostics])
General features
TAT: ca. 1–2 h, random access
+ Rapid, simple to operate, oversimplified workflow (Xpert), accurate, simultaneous or follow‐on rapid DST (except for MTB Ultima), compact, low (Xpert) to minimal (Truenat) infrastructure requirements, and scalable and broad portfolio for other pathogens (Xpert)
− No rapid DST for INH


*Moderate-complexity automated NAATs*
Assay(s)
Abbott RealTi*m*e MTB and RealTi*m*e MTB RIF/INH (Abbott Molecular)
BD MAX MDR‐TB (Becton Dickinson)
cobas MTB and cobas MTB‐RIF/INH (Roche Molecular Systems)
FluoroType MTB (Bruker/Hain Lifescience)
FluoroType MTBDR (Bruker/Hain Lifescience)
General features
TAT: ca. 4–10 h, batched
+ Moderate (BD MAX) to high capacity, accurate, automated except for pretreatment, simultaneous or follow‐on rapid DST for RIF and INH (except for FluoroType MTB), and broad portfolio for other pathogens
− High infrastructure requirements and high capital costs


*Low-complexity manual NAATs*
Assay(s)
Loopamp MTBC Detection Kit (Eiken Chemical)
General features
TAT: ca. 1–2 h, random access
+ Rapid, as simple to operate as low‐complexity automated NAATs, compact, minimal requirements for infrastructure and instrumentation, and easily transportable
− Slightly decreased sensitivity and no rapid DST


*(Near-point-of-care NAATs)*
Assay(s)
(MiniDock MTB [Pluslife])
General features
TAT: ca. 30 min
+ Very rapid, inexpensive, simple to operate, compact, easily transportable, and minimal infrastructure requirements
− Slightly decreased sensitivity and no rapid DST

**Follow-on rapid DST after TB confirmation**


*Low-complexity automated NAATs*
Assay(s)
Xpert MTB/XDR (Cepheid)
General features
TAT: ca. 1–2 h, random access
+ Rapid, oversimplified workflow, compact, and low infrastructure requirements
− Outdated XDR panel (practically a pre‐XDR panel)


*Line-probe assays*
Assay(s)
GenoType MTBDR*plus* (Bruker/Hain Lifescience)
GenoType MTBDR*sl* (Bruker/Hain Lifescience)
Genoscholar NTM + MDR‐TB II (Nipro)
Genoscholar PZA‐TB (Nipro)^a^
General features
TAT: ca. 1–2 days, batched
+ Detection of heteroresistance and low requirements for assay‐specific instrumentation
− Decreased sensitivity, time‐consuming, complex, labor‐intensive, high infrastructure requirements (pre‐ and post‐PCR facilities), and outdated XDR panel (GenoType MTBDRsl)


*Targeted next-generation sequencing tests*
Assay(s)
Deeplex Myc‐TB (GenoScreen/Illumina)
AmPORE‐TB (Oxford Nanopore Technologies)
Tbseq (ShengTing Medical Technology Company)
General features
TAT: ca. 5–48+ h, batched
+ Broad rapid DST panel, high accuracy for MDR and pre‐XDR targets, detection of heteroresistance and mixed infection, NTM detection/identification, strain typing, and rapid (nanopore sequencing)
− Decreased sensitivity for XDR targets; expensive, complex, and labor‐intensive; and high requirements for infrastructure, instrumentation, and expertise

*Note:* New WHO‐recommended assays/categories that are missing from the latest WHO consolidated guidelines document are shown in brackets.

Abbreviations: DR, drug‐resistant; DST, drug susceptibility testing; INH, isoniazid; MDR, multidrug‐resistant; MTB, *Mycobacterium tuberculosis*; MTBC, MTB complex; NAAT, nucleic acid amplification test; PZA, pyrazinamide; RIF, rifampicin; TAT, turnaround time; TB, tuberculosis; XDR, extensively drug‐resistant.

^a^Indirect DST (performed on MTB cultures).

Rapid molecular testing offers substantial advantages over the traditional smear microscopy and culture methods. In addition to species‐specific detection, MDx testing combines the speed of smear microscopy with sensitivity approaching that of culture [8, 9]. Importantly, implementation of one‐sample MDx testing has been associated with decreased airborne isolation compared with three‐sample smear microscopy [13]. Although assay costs and instrumentation requirements still limit accessibility, particularly in low‐resource settings, biosafety considerations are significantly less demanding than for culture [14]. Importantly, the benefits of MDx testing extend beyond pathogen detection. The range of available MDx methods allows simultaneous MTB drug susceptibility testing (DST) or differential diagnostics of NTM. Follow‐on analyses can also include DST for nearly the full spectrum of available anti‐TB drugs, further species identification, and genotyping (e.g., to assess reinfection or mixed infection). This review discusses these methodological aspects of rapid MDx testing for TB, focusing primarily on established procedures and assays included in official guidelines, while also introducing novel approaches that have recently emerged.

## 2. Molecular Detection of TB

Interestingly, most modern MDx TB assays still rely on the MTB‐specific insertion sequence IS*6110*, for which diagnostic potential was suggested already in early 1990 [15], although alternative target sequences have also been employed in some assays, such as the *esx* genes in the Roche cobas MTB assay (Roche Molecular Systems, Pleasanton, California, United States) [16]. However, the genetic organization of IS*6110* is not fixed: Its copy number may vary between strains, and some strains of *Mycobacterium bovis* have been shown to completely lack this sequence [17]. Consequently, most assays target another sequence in addition to IS*6110*, such as *mpb64* in Seegene TB assays (Seoul, Korea), protein antigen B (PAB) in Abbott RealTi*m*e MTB (Abbott Molecular, Des Plaines, Illinois, United States), and IS*1081* by Xpert MTB/RIF Ultra [18–20]. MDx detection of MTB covers other members of the MTB complex. Although differentiation between MTB complex members is possible, it is rarely relevant for clinical decision‐making.

With a limit of detection (LoD) of approximately 10 bacilli/mL of sputum, culture is considered the reference method of TB diagnostics [1]. However, modern MDx TB assays show analytical sensitivities approaching that of culture. For commercial assays, a LoD of 15.6 CFU/mL has been reported for Xpert MTB/RIF Ultra—a second‐generation assay of the original Xpert MTB/RIF assay—and as low as 2.45 CFU/mL for Abbott RealTi*m*e MTB [19, 21]. The low LoD is attributed at least in part to the use of multicopy target sequences. For example, the original Xpert MTB/RIF assay, which targets the single‐copy *rpoB* gene, demonstrated a higher LoD of 112.6 CFU/mL [19]. The claimed LoDs of all WHO‐recommended PCR assays vary between 9 and 30 CFU/mL [9].

MDx methods essentially detect nucleic acids, whereas the specificity of MDx assays in clinical evaluation studies is often compared against the detection of culture‐positive or clinically diagnosed TB. MDx testing has been reported to show higher false‐positivity rates among individuals previously treated for TB, with this rate decreasing over years since treatment completion [22]. Therefore, MDx testing should not be used for treatment monitoring, and test results from patients with a recent history of TB should be interpreted with particular caution. Importantly, such clinically false‐positive cases (detection of TB disease) typically represent analytically true‐positive results (detection of MTB nucleic acid).

## 3. Detection of Pulmonary TB

Rapid MDx TB tests are almost exclusively intended for use with primary or decontaminated sputum samples. By far, most diagnostic accuracy studies and guideline recommendations have focused on the fully automated, low‐complexity Xpert MTB/RIF and Xpert MTB/RIF Ultra assays, which are considered the gold standard methods, whereas fewer studies have been published on other WHO‐recommended assays [23–26]. For the first time, the WHO recommendations on the use of rapid diagnostic assays are class‐specific rather than assay‐specific [8]. However, as evaluation data for assays other than Xpert remain distinctly underrepresented, additional diagnostic accuracy studies are still needed.

Clinical accuracies of the WHO‐recommended assays for detection of pulmonary TB are summarized in Table 2. The presented data compare rapid MDx testing with culture (microbiological reference standard). Interestingly, although comparison with clinical case definition (composite reference standard) decreases sensitivity, specificity is reciprocally increased. A meta‐analysis of Xpert MTB/RIF Ultra demonstrated a 20.8 percentage point (pp) decrease in sensitivity (69.9%; 95% confidence interval [CI]: 52.7%–82.9%) but a 4.0 pp increase in specificity (98.8%; 95% CI: 96.2–99.7) when compared with the composite reference standard instead of culture [23]. These results suggest that culture itself is an imperfect method for TB detection, as it may fail to identify some cases detected by molecular methods.

**Table 2:** Clinical accuracies of the WHO‐recommended assays for detection of pulmonary TB [8].

Test class	Assay	Sensitivity, % (95% CI)			Specificity, % (95% CI)	Reference
		SSM+	SSM−	Total		
Low‐complexity automated NAATs	Xpert MTB/RIF	98 (97–99)	67 (63–72)	85 (82–87)	98 (97–98)	[27]
	Xpert MTB/RIF Ultra	98.8 (98.0–99.3)	80.7 (75.4–85.0)	90.7 (88.2–92.7)	94.8 (92.8–96.3)	[23]
	Truenat MTB Plus	98.3 (96.0–99.4)	76.1 (67.5–84.7)	90.6 (83.7–94.8)	95.7 (94.7–96.5)	[28]
	Pooled	n/a^a^	n/a^a^	90.4 (88.0–92.4)	94.9 (93.0–96.3)	[8]

Moderate‐complexity automated NAATs	Abbott RealTime MTB	99.0 (97.7–100)	88.4 (74.0–95.3)	96.2 (90.2–98.6)	97.1 (93.7–98.7)	[26]
	BD MAX MDR‐TB	100 (89–100)	81 (73–88)	93 (89–96)	97 (96–98)	[26]
	cobas MTB	n/a^a^	n/a^a^	n/a^a^	n/a^a^	
	FluoroType MTB	n/a^a^	n/a^a^	n/a^a^	n/a^a^	
	FluoroType MTBDR	n/a^a^	n/a^a^	n/a^a^	n/a^a^	
	Pooled	n/a^a^	n/a^a^	93.0 (90.9–94.7)	97.7 (95.6–98.8)	[8]

Low‐complexity manual NAATs	Loopamp MTBC	95.9 (92.2–97.9)	59.9 (50.7–68.4)	84.1 (78.3–88.6)	96.1 (94.2–97.4)	[29]

Abbreviations: CI, confidence interval; NAAT, nucleic acid amplification test; SSM, sputum smear microscopy.

^a^Pooled accuracy not available in a published meta‐analysis.

An increasing number of other rapid, fully automated low‐complexity MDx assays for detection of TB are appearing in the market. The STANDARD M10 MDR‐TB assay is currently the only one with peer‐reviewed evaluation reports available. The system is technically analogous to the established GeneXpert system. In both studies reporting its clinical performance, the assay demonstrated accuracy comparable with that of Xpert MTB/RIF Ultra [30, 31], suggesting that this assay may represent a competent alternative to current fully automated low‐complexity tests recommended by WHO.

Moderate complexity automated nucleic acid amplification tests (NAATs) are generally designed for centralized analysis rather than peripheral settings. These methods require more advanced laboratory infrastructure and trained personnel and perform optimally when samples are processed in batches. Diagnostic accuracy is comparable with or even slightly higher than that of Xpert MTB/RIF Ultra (Table 2) [26].

Diagnostic sensitivity is strongly dependent on bacterial load, and study samples that are disproportionately rich in either smear‐positive or smear‐negative cases can substantially affect overall sensitivity. As the analytical sensitivity of smear microscopy is over 5000 bacilli/mL sputum [1], modern in vitro diagnostic (IVD)–certified MDx TB assays should generally show virtually 100% accuracy with smear‐positive samples [26]. Exceptions are usually attributable to technical errors rather than low analytical sensitivity. In contrast, for some assays, low‐positive results have been associated with an increased risk of being false‐positive. The most established example is the semiquantitative “trace” result category of the Xpert MTB/RIF Ultra assay, which is absent in the first‐generation Xpert MTB/RIF assay. Inclusion of this result category modestly reduces assay specificity but substantially increases sensitivity, and vice versa (Table 2) [32, 33]. Of note, due to significantly higher analytical sensitivity compared with smear microscopy, positive MDx results cannot be used as an indicator of high transmissibility.

Unlike for culture, repeated MDx testing is not consistently recommended for the detection of pulmonary TB [8, 9]. However, there appears to be an incremental yield with a two‐sample MDx testing strategy compared with a one‐sample approach. With Xpert MTB/RIF, an increase from 86% (95% CI: 75%–92%) to 92% (95% CI: 84%–96%) has been reported in pooled sensitivity [34].

## 4. Alternative Sample Types for Detection of Pulmonary TB

Detection of pulmonary TB is particularly challenging in individuals and patient groups unable to produce sputum. Specimen types alternative to sputum are generally the same for culture and molecular detection of TB, although only a few MDx TB assays are validated by the manufacturer for analysis of nonsputum sample types. Lower respiratory tract samples other than sputum, bronchoalveolar lavage (BAL) and bronchial washing, are two of the most common sample types [35]. Stool and gastric aspirate, which detect bacilli swallowed from the respiratory tract, as well as induced sputum and nasopharyngeal aspirate, may be used especially with children [8]. In addition to nasopharyngeal aspirate, a range of other upper respiratory tract sample types have been evaluated.

Studies comparing MDx testing with culture using BAL samples have shown both decreased sensitivity (87%, pooled; 95% CI: 84%–90%) and specificity (92%, pooled; 95% CI: 91%–93%) [36]. However, comparison with the composite reference standard significantly increases specificity (98%, pooled; 95% CI: 98%–99%), suggesting excellent potential as a rule‐in test [35]. BAL is more sensitive than bronchial washing and, therefore, the preferable sample of the two whenever possible [35].

Assessing the clinical accuracy of MDx testing with other alternative sample types is more challenging due to substantial variability between studies in the reference methods applied. For instance, MDx analysis of stool may have been compared with gastric aspirate or induced sputum [37]. Consequently, pooled figures compared with microbiological reference standards may not accurately reflect the accuracy of MDx testing compared with culture for these sample types. In addition, technical differences in specific laboratory procedures for challenging sample materials, such as stool, may contribute to inconsistencies in detection rates between studies [37, 38]. This variation likely increases uncertainty and result variability, complicating implementation of local testing procedures. When evaluated against a clinical case definition, pooled sensitivity of 46.5% (95% CI: 29.7%–64.1%) and specificity of 98.4% (95% CI: 91.4%–99.7%) have been reported for Xpert MTB/RIF Ultra with gastric aspirate samples [39]. Similarly, pooled sensitivity of 38% (95% CI: 22%–56%) and specificity of 100% (95% CI: 90%–100%) have been reported for Xpert Ultra with stool samples [37]. For both sample types, use of Xpert MTB/RIF Ultra rather than the original Xpert MTB/RIF assay has been associated with higher sensitivity [37, 40].

Upper respiratory samples have been studied as another alternative sample type for rapid molecular detection of pulmonary TB. This approach in fact encompasses a wide range of sample types using fast and simple sampling techniques such as oral and laryngeal swabs, saliva and mouthwash samples, and nasal/nasopharyngeal samples [41]. Apart from nasopharyngeal aspirate sampling in children and adolescents, there have previously been no WHO recommendations for detection of pulmonary TB using upper respiratory samples [8]. As highlighted in a recent review on oral‐swab sampling for detection of pulmonary TB, the lack of standardized practices can be seen as heterogeneous sampling procedures between studies, including variation in sampling sites (e.g., tongue or buccae), swab types, transport media, sampling frequency, and laboratory procedures [42]. Consequently, results show a wide range from sensitivity of 36%–91% in adults and 5%–42% in children, with overall specificity ranging from 66% to 100% [42]. In a more uniform comparison using laryngeal swab samples with sputum culture as the reference standard, pooled sensitivity was 57.8% (95% CI: 50.5%–65.0%), and specificity was 93.8% (95% CI: 88.4%–96.8%), again with notable variability in accuracy rates between individual studies [41]. Fewer studies have evaluated other upper respiratory sample types for detection of pulmonary TB, but these studies show similar patterns in terms of the range of results and variability in sampling and laboratory procedures (e.g., number of swabs, swab material, and transport media composition) [41].

In a study comparing sampling technique only—specifically, tongue swab versus sputum sampling analyzed with the Xpert MTB/RIF Ultra assay—sensitivity was 77.8% (95% CI: 64.4%–88.0%), and specificity was 100% (95% CI: 97.2%–100%) [43]. No positive results were observed for tongue swab samples when corresponding sputum samples fell into the semiquantitative “very low” or “trace” result categories [43]. Although this approach presents limitations regarding broader applicability in certain patient groups, the study demonstrated that as an easier sampling method in cases where sputum sampling is possible, oral swab sampling is both qualitatively and semiquantitatively less sensitive than sputum sampling, although retaining high specificity.

Interestingly, two recently launched MDx assays are emerging as key contributors to setting a new standard in the field. Truenat MTB Ultima (Molbio Diagnostics, Verna, Goa, India) and MiniDock MTB (MTB Nucleic Acid Test Card, Pluslife, Guangzhou, China) are novel MDx assays intended for use with tongue swabs in addition to sputum. Recent multicountry evaluation studies comparing tongue swab analysis with sputum culture have shown overall sensitivities of 77.9% (95% CI: 70.3%–84.2%) for Truenat MTB Ultima, and 79.6% (95% CI: 73.8%–84.7%) and 85.7% (95% CI: 75.3%–92.9%) for MiniDock MTB [44, 45]. Overall specificities were 98.2% for Truenat MTB Ultima and 99.5%–100% for MiniDock MTB [44, 45]. With Truenat MTB Ultima, tongue swab testing showed a sensitivity 14.9 pp lower than sputum Xpert MTB/RIF Ultra and 18.8 pp higher than sputum smear microscopy [45]. With MiniDock MTB, tongue swab testing showed sensitivities 4.2–6.0 pp lower than sputum swab MiniDock MTB, 8.4–8.7 pp lower than sputum Xpert MTB/RIF Ultra, and 18.3–18.6 pp higher than sputum smear microscopy [44, 45]. The promising results, which exceed the WHO minimum criteria for a nonlower‐respiratory sample (≥ 75% sensitivity and ≥ 98% specificity), have contributed to a major update in the WHO guidelines, which now recommend the use of tongue swabs for initial diagnostic MDx testing in adults and adolescents when lower respiratory tract samples cannot be obtained [10]. Importantly, the recommendations extend beyond the two assays designed for use with tongue swabs. By increasing sampling uniformity, the new assays and recommendations are likely to significantly facilitate the implementation of alternative sampling procedures for improved detection of pulmonary TB.

## 5. Detection of Extrapulmonary TB

Although TB is primarily a pulmonary disease, extrapulmonary TB accounts for approximately 20% of all active cases of TB [12]. Extrapulmonary TB is more common in, but not limited to, high‐risk groups with immature or weakened immunity such as children, the elderly, and immunocompromised individuals [46]. In late HIV infection, extrapulmonary TB may account for 50% of all TB cases [47, 48]. Children under the age of 5 have traditionally been considered the most vulnerable subpopulation for developing a disseminated, fulminant disease, often associated with TB meningitis [48, 49]. However, in low TB prevalence, high‐income settings, the elderly may carry the highest risk of extrapulmonary TB [48, 50]. Lymph nodes (50%) and pleura (18%) are the tissues most commonly affected, although extrapulmonary TB can involve virtually any tissue, including the genitourinary system (13%), bones and joints (6%), the gastrointestinal system (6%), the central nervous system (3%), and the spine (3%) [46, 48]. In approximately 5% of TB cases in immunocompetent individuals, both lungs and extrapulmonary sites are affected, whereas in immunocompromised patients, this proportion can reach 50% [47]. Miliary TB is observed in about 20% of extrapulmonary TB cases but represents only about 2% of all TB cases [47]. These findings underscore the importance of considering different sample types and high‐risk populations for a comprehensive assessment of TB diagnostics.

Evaluation of MDx assays for the detection of extrapulmonary TB is complicated by limitations of the microbiological reference method, mycobacterial culture. Reported culture sensitivities are 60%–70% for lymph node aspirates, 30%–50% with pleural fluid, and only around 30% for cerebrospinal fluid (CSF) [51]. Although extrapulmonary TB is clinically significant, only a few commercial MDx assays have been validated by the manufacturer for use with nonsputum sample types, the Seegene MTB product family being one of the most established exceptions [52]. However, the limited number of clinical studies evaluating Seegene assays for detection of extrapulmonary TB typically reports pooled performance values, combining all extrapulmonary sample types into a single category [52–54]. Since the specimen type has a major influence on clinical performance in detection of extrapulmonary TB, this approach has only limited relevance. The second‐generation Truenat MTB assay, Truenat MTB Plus (Molbio Diagnostics), is a recently launched MDx assay intended for detection of extrapulmonary TB. Studies of the original Truenat MTB assay suggest clinical accuracy somewhat inferior to Xpert MTB/RIF Ultra, whereas studies of the Truenat MTB Plus assay demonstrate comparable or even superior accuracy relative to Xpert MTB/RIF Ultra [25, 55–58].

Interestingly, Xpert MTB assays are the most widely evaluated MDx assays for use with extrapulmonary samples, despite not being validated for such use by the manufacturer. In fact, until 2025, these assays were the only ones recommended by WHO for detection of extrapulmonary TB [8, 16]. More specifically, in adults and children suspected of extrapulmonary TB, WHO recommends MDx testing in CSF, lymph node aspirate or biopsy, pleural fluid, peritoneal fluid, pericardial fluid, and synovial fluid samples. Clinical accuracies of the initial Xpert MTB assays with different extrapulmonary sample types are summarized in Table 3.

**Table 3:** Clinical performance of initial Xpert MTB assays with extrapulmonary samples [25, 59].

Specimen type	Reference standard	Xpert MTB/RIF		Xpert MTB/RIF Ultra	
		Sensitivity, % (95% CI)	Specificity, % (95% CI)	Sensitivity, % (95% CI)	Specificity, % (95% CI)
Cerebrospinal fluid	MRS	71.1 (62.8–79.1)	96.9 (95.4–98.0)	88.2 (83.7–91.6)	96.0 (86.8–98.9)
	CRS	42.3 (32.1–52.8)	99.8 (99.3–100.0)	60.3 (80.9–69.0)	99.2 (98.1–99.7)
Pleural fluid	MRS	49.5 (39.8–59.9)	98.9 (97.6–99.7)	74.0 (60.8–83.9)	88.1 (78.8–93.6)
	CRS	18.9 (11.5–27.9)	99.3 (98.1–99.8)	43.6 (32.8–55.0)	99.2 (95.2–99.9)
Pleural tissue	MRS	0–85^a^	97–100^a^	80–100^a^	75–86^a^
	CRS	0 (0–23)	98 (87–100)	54–81^a^	94–97^a^
Lymph node aspirate	MRS	59–100^a^	57–100^a^	85.3 (73.4–92.4)	74.1 (63.5–82.5)
	CRS	81.6 (61.9–93.3)	96.4 (91.3–98.6)	71.3 (64.3–77.4)	97.4 (82.3–99.7)
Lymph node tissue	MRS	82.4 (73.5–89.7)	80.3 (60.3–91.5)	96.5 (84.7–99.3)	79.4 (65.4–88.8)
	CRS	33–76^a^	85–100^a^	61.5 (47.1–74.2)	96.7 (91.5–98.7)
Urine	MRS	85.9 (71.4–94.3)	98.1 (93.1–99.7)	100^a^	28–100^a^
	CRS	33–41^a^	100^a^	13–34^a^	95–100^a^
Bone or joint aspirate	MRS	97.9 (93.1–99.6)	97.4 (80.2–100.0)	96.6 (87.2–99.1)	91.1 (80.8–96.2)
	CRS	82–94^a^	100^a^	96 (91–99)^a^	97 (85–100)^a^
Bone or joint tissue^b^	MRS	50–100^a^	94–100^a^	—	—
Peritoneal fluid^b^	MRS	59.1 (42.1–76.2)	97.6 (95.4–98.9)	33–67^a^	94–100^a^
Peritoneal tissue^b^	MRS	50 (7–93)^a^	92 (73–99)^a^	—	—
Pericardial fluid^b^	MRS	61.4 (32.4–82.4)	89.7 (74.9–99.0)	84.0 (73.9–90.7)	86.6 (79.5–91.5)
Blood^b^	MRS	56 (21–86)^a^	94 (85–98)^a^	38 (33–43)^a^	98 (94–100)^a^

Abbreviations: CI, confidence interval; CRS, composite reference standard; MRS, microbiological reference standard.

^a^No meta‐analysis performed.

^b^No studies evaluated against CRS.

As with pulmonary samples, the lower specificity of the Xpert MTB/RIF Ultra assay is primarily associated with the “trace” result category, for which no consistent clinical interpretation has been established in the diagnosis of extrapulmonary TB [60–62]. Notably, assay specificities are considerably higher when PCR is compared with a composite reference standard rather than culture. This further supports the view that MDx assays are reliable rule‐in tests, complementing culture by increasing diagnostic yield, but also decreasing time‐to‐treatment [63, 64]. As with pulmonary samples, analysis of smear‐positive samples has a virtually 100% accuracy [63].

In general, the analysis of extrapulmonary samples with commercial MDx assays follows protocols similar to those used for pulmonary samples. However, there is a considerable potential for variation in sample preparation. For example, concentrating CSF samples has been shown to significantly increase sensitivity (88.4%–90.5% with Xpert MTB/RIF Ultra) and specificity (88.6%–91.9% with Xpert MTB/RIF Ultra) [26].

Both culture and MDx testing have reduced sensitivity in PLHIV. Urine lateral flow lipoarabinomannan (LAM) tests, recommended by WHO for detection of TB in PLHIV across all age groups, represent a distinct diagnostic approach in this patient subgroup [8]. With relatively nonoverlapping detection of TB, the combined use of LAM and MDx tests has been shown to increase diagnostic yield in both detection of pulmonary and extrapulmonary TB in PLHIV [65, 66].

## 6. Rapid Drug Susceptibility Testing

Rapid drug susceptibility testing (DST) represents another important area of molecular TB diagnostics. This is particularly relevant with drug‐resistant TB being one of the key challenges for effective TB control. Some applications, such as the Xpert MTB/RIF Ultra and BD MAX MDR‐TB (Becton Dickinson, Franklin Lakes, United States) assays, combine rapid DST analysis with MTB detection in a single test, whereas some others, such as the cobas MTB and Abbott RealTi*m*e MTB assays, involve a separate reflex assay for rapid DST [16]. In addition to real‐time PCR assays, WHO also endorses more complex and labor‐intensive DNA‐hybridization line probe assays as follow‐on tests for confirmed MTB‐positive cases [8].

Rapid DST methods involve targeted nucleic acid amplification, which inherently imposes limitations on analytical coverage. Whereas phenotypic, culture‐based DST detects resistance regardless of the underlying mechanism, a rapid DST assay must be specifically designed to capture all genetic factors contributing to resistance against a given drug in order to provide reliable results. This limitation is primarily attributed to the design of oligonucleotides, that is, the selection of primer and probe sequences.

In many cases, commercial MTB rapid DST assays involve detection of both wild‐type and mutant sequences. In real‐time PCR assays, this is commonly achieved by using melting curve analysis to differentiate wild‐type sequences (susceptible) from mutant sequences (resistant), that is, by detecting the presence or absence of mutations [16]. For instance, the Xpert MTB/XDR assay employs a sloppy molecular beacon probe technology, in which optimal hybridization and the corresponding melting peak distinguish wild‐type sequences from suboptimally hybridizing mutant sequences [67]. This approach, based on specific–nonspecific hybridization, can cover a wide range of resistance‐conferring mutations. For instance, although most resistance‐conferring mutations are single‐nucleotide polymorphisms, any mutation within the 81‐base‐pair RIF resistance‐determining region (RRDR) of the *rpoB* gene is assumed to confer RIF resistance [68]. This approach has an important implication for result interpretation. Detection of MTB is often based on multicopy targets such as IS*6110*, whereas rapid DST targets single‐copy genes such as *rpoB*. Consequently, there is a risk of detecting the MTB target while failing to detect the rapid DST target. In such cases, assays employing melting curve analysis report the DST result as indeterminate (unable to define target as susceptible or resistant) to prevent a false‐susceptible interpretation. As with MTB detection, MDx assays may have distinct sensitivities for detecting DST targets, which may be seen as differences in indeterminate DST result rates [30]. For example, the claimed LoDs for DST analysis with the Abbott RealTi*m*e and cobas MTB assays are 3 and 20 times the LoD for MTB detection, respectively [9].

Commercial MTB rapid DST assays are primarily divided into MDR/RR‐TB assays, which detect resistance to RIF (*rpoB*) only, or RIF and isoniazid (INH; *inhA* and *katG*), and XDR‐TB assays (extensively drug‐resistant TB), which detect resistance to fluoroquinolones (*gyrA* and *gyrB*) and second‐line injectable agents amikacin (*eis* and *rrs*), kanamycin (*eis* and *rrs*), capreomycin (*eis*) (assay nomenclature according to previous XDR‐TB definition) [16, 69]. In general, MTB rapid DST for primary targets RIF and INH is highly accurate compared with culture‐based phenotypic DST [8]. For RIF, mutations in the RRDR have been reported to account for more than 95% of all RIF resistance [9, 48]. For INH, resistance mechanisms are more complex. Most assays detect mutations in the *katG* gene and *inhA* promoter region, but mutations in the *ahpC-oxyR* intergenic region and *fabG1* gene also confer resistance [48, 67, 70].

Seegene′s XDR‐TB assay, analogous to the abovementioned MDR‐TB assay, was the only widely available option to the WHO‐recommended GenoType MTBDRsl (Bruker/Hain Lifescience, Nehren, Germany) for rapid second‐line DST until Cepheid launched the Xpert MTB/XDR assay in 2020, which was soon also recommended by WHO [16, 67, 69, 71]. Although the assay can be used as a stand‐alone test, MTB detection is based on a single‐copy sequence target, and therefore, the assay is intended as a follow‐on test for MTB‐positive samples [67]. Indeed, the assay covers detection of INH resistance in addition to the “traditional” (pre‐)XDR‐TB targets, further reflecting its design as a supplementary follow‐on test to the Xpert MTB/RIF (Ultra) assay. In addition to the common targets *inhA* and *katG* for INH resistance, Xpert MTB/XDR is the only WHO‐recommended assay to also target mutations in the *oxyR-ahpC* intergenic region and *fabG1* gene, providing increased coverage for detection of INH resistance [67]. The clinical accuracy of the assay has been shown to be virtually identical with the GenoType MTBDR*plus*/*sl* assays, with sensitivities of 93%–95% for isoniazid and fluoroquinolones, 86% for kanamycin, 73% for amikacin, 61% for capreomycin, and identical specificities of at least 98% for each drug [72]. As reflected by the data, rapid DST based on sequence targets *gyrA* and *gyrB* is highly accurate for detecting fluoroquinolone resistance, whereas detection of resistance against second‐line injectable agents remains more complex and challenging [68, 72]. The Xpert MTB/XDR assay targets the *inhA* promoter region as a dual indicator for both INH and ethionamide resistance, a feature not applied by other pre‐XDR‐TB assays [67]. However, a significant proportion of known mutations associated with ethionamide resistance are related to *ethA* [68]. This likely explains the relatively poor sensitivity of Xpert MTB/XDR for detection of ethionamide resistance, resulting in a high rate of false‐susceptible results.

WHO updated the definitions of drug‐resistant TB in 2020. In the update, the previous XDR‐TB definition was in practice downgraded to pre‐XDR‐TB (MDR‐TB with fluoroquinolone resistance), whereas XDR‐TB was assigned a new definition (pre‐XDR with bedaquiline or linezolid resistance) [73]. Therefore, most currently available commercial XDR‐TB‐labeled MDx assays are still based on the outdated definition, detecting pre‐XDR‐TB instead of XDR‐TB. Additionally, such assays typically detect resistance to agents no longer recommended for TB treatment by WHO for TB treatment, namely, kanamycin and capreomycin [67, 69, 74]. FluoroType MTB/XDR is a novel MDx assay within the Bruker/Hain Lifescience FluoroType product family and can be used directly as a follow‐on test for the WHO‐recommended FluoroType assays. Drugs covered by the assay include linezolid, a drug conferring current‐definition XDR‐TB, as well as other WHO‐recommended drugs, fluoroquinolones, amikacin, and ethambutol. The recently launched assay is not yet recommended by WHO but meets the WHO minimum target product profile criteria for rapid DST of fluoroquinolones [75]. Currently, the only commercial applications capable of detecting MTB resistance to both bedaquiline and linezolid are targeted next‐generation sequencing (tNGS) applications [8]. Correspondence of available rapid DST panels with currently recommended standard drug regimen agents is presented in Table 4.

**Table 4:** Comparison of currently recommended standard anti‐TB drug regimens with drug susceptibility targets of available MDx assays [8, 9, 76].

Diagnostics									Drug treatment				Epidemiology
Drug	RR‐TB assays^a^	MDR‐TB assays^b^	Xpert MTB/XDR	GenoType MTBDR*sl*	(FluoroType MTB/XDR)	Deeplex Myc‐TB	AmPORE‐TB	Tbseq	Drug	DS‐TB^d^	Hr‐TB	MDR/RR‐TB^d^	Drug resistance
Rifampicin	x	x				x	x	(x)	Rifampicin^c^	x	x		RR/MDR
Isoniazid		x	x			x	x	(x)	Isoniazid	x			Hr/MDR
Pyrazinamide						x	(x)	(x)	Pyrazinamide	x	x	x (2/4)	
Ethambutol					(x)	x	(x)	x	Ethambutol	x (1/2)	x		
Fluoroquinolones			x	x	(x)	x	x	(x)	Fluoroquinolones	x (1/2)^e^	x	x^e^	Pre‐XDR
Amikacin			x	x	(x)	x	x	(x)					
(Kanamycin)			(x)	(x)		(x)	(x)	(x)					
(Capreomycin)			(x)	(x)		(x)	(x)	(x)					
Ethionamide			x			x	(x)	(x)					
Linezolid					(x)	x	x	(x)	Linezolid			x	XDR^f^
Bedaquiline						x	(x)	(x)	Bedaquiline			x	XDR^f^
Clofazimine						x	(x)	(x)	Clofazimine			x (2/4)	
Streptomycin						x	x	(x)					
Delamanid							(x)		Delamanid			x (1/4)	
Pretomanid							(x)		Pretomanid			x (1/4)	
PAS								(x)					
Cycloserine								(x)					

*Note:* Drugs, assays, or rapid DST targets in brackets are no longer (kanamycin and capreomycin) or not yet (others) recommended by WHO.

Abbreviations: DS, drug‐susceptible; Hr, isoniazid‐monoresistant; MDR, multidrug‐resistant; PAS, para‐aminosalicylic acid; RR, rifampicin‐resistant; TB, tuberculosis; XDR, extensively drug‐resistant.

^a^Xpert MTB/RIF, Xpert MTB/RIF Ultra, and Truenat MTB‐Rif Dx.

^b^Abbott RealTi*m*e MTB RIF/INH, BD MAX MDR‐TB, cobas MTB‐RIF/INH, FluoroType MTBDR, and GenoType MTBDR*plus.*

^c^Or rifapentine, regimen‐specific.

^d^If not included in all standard regimens within the class (DS‐TB or MDR/RR‐TB), the number of associated regimens is indicated in brackets.

^e^Either levofloxacin or moxifloxacin, regimen‐specific.

^f^Resistance against at least bedaquiline or linezolid.

Although the sensitivity and specificity of first‐line rapid DST are high, concerns for false RIF‐resistant results have been reported. As a theoretical example, with a 1% prevalence of resistance, 100% assay sensitivity, and 99% specificity, the positive predictive value of resistance detection already falls to 50%. In a recent study conducted in Rwanda, 54 out of 101 (53%) cases initially tested as RIF‐resistant with Xpert MTB/RIF Ultra were later confirmed as false‐resistant by reference methods (phenotypic DST and/or sequencing) [77]. False‐resistant results were primarily associated with samples with low bacterial load: 91% of the false‐resistant cases were within the “very low” result category, whereas the remaining 9% were within the “low” category [77]. Overall, 89% of all resistant results in the “very low” category were false‐resistant [77]. Similar findings have been observed with the predecessor Xpert assay [78]. Although the low bacterial load is suggestive of technical failures due to signal intensity below the positivity threshold, false rapid DST results may also arise from heteroresistance or mixed infection, mutations not covered by the rapid DST assay, or silent mutations. Fully automated rapid DST methods are not designed to detect mixed infection or heteroresistance. At best, such cases are reported as resistant, although in some cases, mixed probe hybridization patterns may be suggestive of heteroresistance [79]. In contrast, line probe assays, which are based on manual result interpretation, allow more explicit—although not comprehensive—detection of heteroresistance [79]. Different assays may have distinct thresholds for detecting resistance in mixed infections. For example, at least 20%–80% of MTB cells must be resistant to be detected as resistant by Xpert MTB assays, whereas the threshold for GenoType MTBDRplus is lower, 5%–10% [79]. Mutation coverage is an issue inherent to any targeted MDx method. For instance, the Ile491Phe mutation is located outside the *rpoB* core region and is, therefore, not detected by first‐line WHO‐recommended assays [80]. Strains carrying this mutation have spread silently and caused outbreaks of drug‐resistant TB [80]. Finally, when melting curve analysis is based on complete probe hybridization with wild‐type sequence and incomplete hybridization with mutant sequences, mutations that do not confer resistance may still be interpreted as resistant by rapid DST assay [81].

Of note, sample type does not appear to affect the accuracy of direct rapid DST per se, as high sensitivity and specificity are retained with extrapulmonary samples [25, 63]. However, extrapulmonary sample types often have very low bacterial loads [82], which may be associated with a higher rate of indeterminate rapid DST results (MTB detected and DST analysis failed) and a higher risk of false‐resistant results.

### 6.1. Targeted Next‐Generation Sequencing

Targeted next‐generation sequencing (tNGS) is a technology only recently introduced in commercial TB diagnostics. The application combines features of NGS and multiplex PCR workflows, such as instrumentation, workflow, and the ability to determine nucleic acid sequences (NGSs), and, on the other hand, analysis of specific genomic regions, providing qualitative results for predefined analytes (multiplex PCR). The main differences from whole‐genome sequencing are related to genomic coverage and, in commercial assays, the inclusion of a predefined workflow for laboratory procedures, a data analysis pipeline, and standardized result reporting.

Since 2024, the WHO has recommended tNGS assays for rapid DST of MTB, primarily as follow‐on tests for first‐line WHO‐recommended tests [83]. The recommended assays include Deeplex Myc‐TB (Genoscreen, Lille, France), AmPORE‐TB (Oxford Nanopore Technologies, Oxford, United Kingdom), and TBseq (Hangzhou ShengTing Medical Technology Co., Hangzhou, China) [8]. Importantly, tNGS assays are the only WHO‐recommended MDx methods to detect resistance to all standard first‐line regimen drugs and XDR‐TB‐determining drugs [8]. In addition to rapid DST, all WHO‐recommended assays provide mycobacterial species identification and MTB strain typing features for differential diagnostics and detection of mixed infections, thereby covering most of the mycobacterial diagnostic spectrum in a single assay.

The traditional limitations of NGS methods are related to high costs, substantial requirements for expertise and infrastructure, and slow turnaround times. However, with predefined workflows, commercial tNGS assays are designed for relatively straightforward implementation, with analysis costs approaching those of other high‐end MDx assays [84, 85]. The cost‐efficiency of tNGS methods depends on factors such as instrumentation, use of tNGS for diagnostics of multiple diseases, number of samples per run, and accessory follow‐on TB analyses [86]. Most notably, routine whole‐genome sequencing of isolated strains is, in most cases, almost fully redundant with tNGS analysis. When assessing impacts on healthcare and economic costs, such differences and adaptations to local practices have been shown to qualitatively affect the cost‐efficiency of implementing tNGS in TB diagnostics [86]. However, findings from cost‐efficiency analyses may vary substantially from setting to setting. For example, according to a recent TBNet report, the costs per standard MDR‐TB drug regimen in Europe in 2021 ranged from approximately EUR 500 to 41,000 [87]. In comparison, reported examples of tNGS unit costs (Deeplex Myc‐TB) ranged between $121 and $257 [8].

Nanopore sequencing (Oxford Nanopore Technologies) offers a distinct alternative to traditional NGS. This platform enables a simplified workflow using a portable, low‐cost, pocket‐sized instrument (MinION platform) with real‐time analysis. Compared with Deeplex tNGS, nanopore sequencing has been shown to be able to reduce turnaround times from a few days to as little as half a day [88]. Notably, tNGS testing may demonstrate longer turnaround times when applied under routine diagnostic conditions instead of optimal conditions [89].

In patients with bacteriologically confirmed pulmonary TB, tNGS is associated with pooled sensitivities of > 95% for isoniazid, moxifloxacin, and ethambutol, over 93% for RIF and levofloxacin, and 88% for pyrazinamide [8]. Sensitivity in confirmed RIF‐resistant cases is slightly higher for first‐line drugs and fluoroquinolones, over 95% for streptomycin, 87% for amikacin, and around 70% for bedaquiline, linezolid, and clofazimine [8]. Pooled specificity is uniformly high (over 95%) for all drugs but streptomycin (75%) [8]. Somewhat paradoxically, the sensitivity and specificity for detecting RIF resistance appear lower with tNGS than with first‐line WHO‐recommended tests, despite the broader genomic coverage of tNGS assays [8]. However, when compared with the WHO MTB mutation catalogue, sensitivity of tNGS is comparable [8, 68]. In fact, only two resistance‐conferring mutations have been annotated outside the RRDR, *rpoB* Val170Phe and Ile491Phe [68]. If these specific mutations are absent in the local setting, RIF‐DST with the WHO‐recommended first‐line assays and tNGS assays is similarly based on mutations in the RRDR: first‐line assays detecting any mutations in the RRDR and tNGS assays detecting annotated mutations and nonsilent mutations.

The WHO Catalogue of mutations in MTB complex and their association with drug resistance is an extensive database for genomic DST interpretation. The first version of the catalogue was updated in 2023, increasing the number of mutations associated with resistance, particularly for new and repurposed second‐line agents such as bedaquiline, linezolid, clofazimine, and delamanid [68]. Consequently, tNGS studies based on the first version of the catalogue may show slightly lower sensitivities than those based on the updated version. Following a recent call for data, a third edition of the mutation catalogue is anticipated.

Importantly, although the WHO‐recommended tNGS assays may appear to target the same genomic regions for DST, the genomic coverage of different assays is not fully overlapping. In other words, different assays may have unique caveats in the mutations covered by the analysis [88].

Apart from genomic coverage, tNGS has been reported to give higher levels of indeterminate DST results at low bacterial loads [90]. Operator‐dependent factors have also been suggested as possible contributors to issues in tNGS results, including both indeterminate and discordant DST results [90]. In this sense, the quality of nucleotide‐level tNGS results seems to be more operator‐dependent compared with the more imprecise PCR melting curve analysis. Again, it should be noted that although the presence of indeterminate DST results decreases the cost‐efficiency of tNGS analysis, it prevents the reporting of false DST results. In general, tNGS is considered at least as sensitive as Xpert MTB/RIF both for valid (nonindeterminate) rapid DST analysis and MTB detection [91–94]. No studies were found comparing tNGS with other, more sensitive first‐line WHO‐recommended tests.

In terms of heteroresistance and mixed infection, tNGS is superior to other WHO‐recommended MDx methods. Although its sensitivity for detecting resistance in such cases has been reported to be similar to that of line probe assays, tNGS explicitly identifies and quantifies strains and mutations present in the sample [79].

## 7. Nontuberculous Mycobacteriain TB Diagnostics

In many settings, TB diagnostics may in fact correspond to diagnostics of mycobacteria. Nontuberculous mycobacteria (NTM) collectively refer to mycobacterial species other than MTB or *Mycobacterium leprae*, the causative agent of leprosy, and include nearly 200 valid species [95]. NTM may cause pulmonary disease that is clinically indistinguishable from TB [96]. Detection is covered by traditional diagnostic methods—isolates can be subsequently differentiated from MTB or identified by other methods. An increasing number of MDx assays combine species‐ or genus‐specific detection of mycobacteria with detection of MTB. Such assays may offer broad coverage similar to traditional methods while enabling essential differentiation between the obligate pathogen MTB and the opportunist NTM. Importantly, these assays do not generally include rapid DST analysis for MTB (or NTM).

The clinical isolation of NTM may reflect sample contamination, transient or persistent colonization of the airways, or a causative agent of disease [97]. Nonetheless, NTMs are increasingly associated with disease and contribute to considerable morbidity worldwide. Particularly in developed countries, incidence of NTM pulmonary disease (NTM‐PD)—the most established manifestation—has risen and may exceed the decreasing incidence of TB, although this trend is at least partially attributable to increased awareness of NTM infections and greater diagnostic capacity [96, 98]. Only a limited number of NTM species account for the majority of infections, although many more are capable of causing clinical disease [97, 99]. *Mycobacterium avium* complex (MAC) members, *Mycobacterium abscessus*, *Mycobacterium kansasii*, *Mycobacterium malmoense*, and *Mycobacterium xenopi* are among the species most frequently associated with disease [97, 100]. In addition to causing infections that may be misdiagnosed as TB, NTMs have also been shown to coinfect individuals with TB [101].

Codetection of MTB and NTM can be divided into two distinct strategies. Assays intended for use with primary samples often employ a duplex approach, targeting an MTB‐specific sequence such as IS*6110*, and a pan‐mycobacterial sequence such as 16S rRNA or *rpoB* to differentiate MTB and NTM [20, 30]. Detection of MTB is based on the presence of the MTB target irrespective of the pan‐mycobacterial target, whereas detection of NTM is based on the presence of the pan‐mycobacterial target in the absence of the MTB target [20, 30]. The other strategy employs a multiplex approach, targeting species‐specific sequences for various in‐panel species and is more often associated with analysis of positive cultures [102–105].

The duplex assays are typically real‐time PCR assays intended for detecting MTB and NTM in clinical, primarily respiratory specimens. Although an increasing number of such assays are available on the market, only a handful of peer‐reviewed studies exist, most of which involve evaluation of the Seegene MTB/NTM assays. Different studies report a wide range of sensitivities: 61%–100% with smear‐positive samples and 4%–41% with smear‐negative samples [20, 54, 106]. Importantly, all studies reported MTB sensitivity below 100% with smear‐positive samples [20, 54, 106]. STANDARD M10 MTB/NTM is the first low‐complexity assay (fully automatic, random‐access, and point‐of‐care compatible) for MTB/NTM co‐detection. This assay has been shown to perform comparably with the Xpert MTB/RIF Ultra assay for MTB detection while also increasing the yield in NTM detection compared with sputum smear microscopy [30]. Individual evaluation studies of other commercial assays (GeneDia, Advansure, EZplex, PowerChek) have been conducted, all in Korea [48, 107–109]. Regardless of detection accuracy, these studies uniformly report an adequate level of clinical accuracy. This may be attributed to the added value of NTM detection in clinical settings where NTM would not be routinely examined. Evaluation of clinical performance should be conducted locally, as the geographical distribution of NTM species varies substantially, and MDx assays may have distinct sensitivities for different NTM species [20, 30, 110]. It is notable that detection of NTM as a group of species provides relatively little information for clinical decision‐making, even with respect to differential diagnosis between TB and NTM‐PD; detection of NTM should be accompanied by subsequent species identification to further assess possible clinical significance [97].

## 8. Novel Molecular Approaches in Rapid TB Diagnostics

### 8.1. Alternatives to Real‐Time PCR

The WHO guidelines for initial MDx testing for TB are dominated by real‐time PCR assays. One exception is the Loopamp MTBC Detection Kit (Eiken Chemical, Tokyo, Japan), which is based on loop‐mediated isothermal amplification (LAMP) technology [8]. For isothermal amplification, a simple heat block is required instead of a thermal cycler, and detection is based on visual inspection—under a UV light in the case of the Loopamp MTBC assay [16]. Although involving more manual steps than the automated WHO‐recommended assays—therefore classified as a low‐complexity manual NAAT by the WHO—the independence from specific instrumentation may increase its feasibility in peripheral and/or low‐resource settings. The novel MiniDock MTB Test, based on RNase hybridization–assisted amplification (RHAM) technology, is another commercial system for isothermal detection of MTB [44]. The system requires some assay‐specific instrumentation but is battery‐operated and has minimal costs compared with other WHO‐recommended MDx systems. Subsidized costs are $330 for instrumentation and $3.60 per test [111]. The MiniDock MTB test has recently been recommended by WHO and is the first product in the novel class of near‐point‐of‐care NAATs, intended for use in decentralized, nonlaboratory settings. Both Loopamp MTBC and MiniDock MTB are used only for detection of TB, as neither test includes simultaneous or follow‐on rapid DST analysis.

Several other isothermal amplification techniques, including strand displacement amplification (SDA), helicase‐dependent amplification (HAD), recombinase polymerase amplification (RPA), and rolling circle amplification (RCA), have also been studied and evaluated in TB diagnostics. Although the Loopamp MTBC and MiniDock MTB tests have somewhat lower diagnostic accuracies compared with WHO‐recommended PCR assays, some experimental isothermal amplification applications have shown LoDs lower than those of any WHO‐recommended assays [112].

Clustered regularly interspaced short palindromic repeats (CRISPR) technology, a widely established technology in gene technology, has been increasingly studied as a means to improve accessible diagnostics of infectious diseases, including TB. Although there are currently no commercial CRISPR assays for TB diagnostics, experimental assays have been reported to have pooled sensitivity of 93% (95% CI: 85%–99%) and specificity of 97% (95% CI: 94%–99%), comparable with the accuracies of the current WHO‐recommended assays [113]. Diagnostic CRISPR‐Cas systems involve specific detection of a target sequence, which is followed by nonspecific amplification of a reporter signal and preceded by a preamplification step to increase sensitivity [114]. However, dependence on preamplification also increases process complexity, and true point‐of‐care applications are likely to await the development of preamplification‐free methods [114].

### 8.2. Digital PCR

Digital PCR is a novel, third‐generation PCR method for sensitive detection and absolute quantification of nucleic acids. Consequently, the applicability of digital PCR has been studied in areas testing the limits of routine diagnostics. In terms of diagnostic accuracy, digital PCR has been shown to perform exceptionally well with paucibacillary sample types such as pleural effusion and CSF, demonstrating superior sensitivity to Xpert MTB/RIF and culture [115, 116]. However, all studies involving Xpert analysis compared multicopy sequence target digital PCR (IS*6110*/IS*1081*) with the single‐copy sequence target Xpert MTB/RIF assay (*rpoB*) [115–118]. The superior discriminatory power of digital PCR makes it an excellent tool for detecting heteroresistance. Studies have shown its ability to detect resistant variants at thresholds below 1%, which is lower than with any other MDx method [119, 120]. Owing to its intrinsic capability for absolute quantification, digital PCR has also been shown to aid treatment monitoring [117].

Digital PCR applications do not align with common real‐time PCR processes, as they involve distinct workflows, specialized instrumentation, and a high level of expertise. These characteristics increase their overall costs [121]. Turnaround time is relatively long (days instead of hours), whereas capacity remains relatively low [121]. Currently, there are no commercial digital PCR assays for TB diagnostics. Although digital PCR shows certain advantages in specific, difficult‐to‐diagnose cases, its relevance mainly lies in research, whereas its feasibility in routine diagnostics remains limited.

### 8.3. Metagenomics

Metagenomics offers the broadest coverage among diagnostic analysis methods, representing a so‐called hypothesis‐free approach with a microbial detection yield higher than that of any other single diagnostic method. Metagenomics is based on NGS technology (mNGS) and employs an approach that detects—depending on the focus—any nucleic acid present in the sample. For example, diagnostic applications may involve detection of any bacterial, viral, and fungal DNA. Notably, accurate mNGS in clinical microbiology is highly dependent on effective host DNA depletion [122].

Due to the immense amount of data produced in a single analysis, mNGS results are complex to interpret: Detected microbes may include contaminants, commensals, and dead organisms, as well as unexpected pathogens not covered by conventional methods [123]. Other limitations align with those of tNGS methods. The main benefits are particularly related to the detection of atypical pathogens, which may be difficult to detect or not captured by conventional methods such as culture [124]. With respect to TB, mNGS approaches have been shown to improve diagnostics by revealing previously undetected cases and mixed infections, potentially influencing overall treatment strategies [125–128].

Perhaps surprisingly, several studies have reported mNGS diagnostic accuracies comparable with Xpert MTB/RIF [129]. These results are promising for the potential use of mNGS approaches in primary diagnostics. However, there are no standardized methods for TB diagnostics by mNGS, and diagnostic performance may vary from protocol to protocol [122]. Notably, no studies have compared mNGS with the more sensitive Xpert MTB/RIF Ultra assay. Although the process is somewhat similar to tNGS, the lack of standardization and absence of commercial assays substantially increases the demand for laboratory and bioinformatics expertise, which decreases the overall feasibility of the method [123].

Although mNGS diagnostics may not be cost‐efficient in primary TB diagnostics, current literature suggests that such approaches can directly (through positive detection) or indirectly (through differential diagnosis) complement routine TB diagnostic workflows, especially in severe or complex cases such as CNS infections, infections in immunocompromised individuals, and nonresolving lower respiratory tract infections [124, 130–132].

### 8.4. Indirect TB Detection Methods

As presented previously, molecular detection of TB traditionally involves direct detection of MTB nucleic acid. Even with point‐of‐care‐ready applications available, this approach is not considered sufficient to fill diagnostic gaps, which are mainly attributed to limited accessibility due to centralized services, complex care pathways, and insufficient clinical applicability with children, PLHIV, and extrapulmonary TB [133, 134]. For this reason, the 2014 WHO meeting report identified a rapid biomarker‐based, nonsputum‐based test and a community‐based triage or referral test as high‐priority target product profiles for new TB diagnostics [135]. Triage tests that can be used by first‐contact healthcare providers could reduce diagnostic and treatment delays, as well as overall costs, by simplifying initial screening and increasing accessibility [136]. Importantly, such assays would not be dependent on the challenges associated with obtaining optimal sputum samples.

Transcriptome analysis has been widely used in TB research as a tool to study and understand the complex infection biology between the host and the pathogen. In TB diagnostics, this approach has been employed to map TB‐specific host gene expression profiles. Different studies have identified such transcriptional signatures ranging from a few to several tens of genes to discriminate active TB disease from latent TB infection, other respiratory diseases, or healthy controls [137, 138]. Perhaps unsurprisingly, the associated genes are primarily interferon‐inducible, along with other genes involved in immune function [137, 139]. With varying performance, some of these transcriptional signatures have been reported to achieve clinical performance exceeding the WHO minimum criteria for a triage test for TB—i.e., at least 90% sensitivity and 70% specificity compared with the confirmatory test for pulmonary TB [138].

With respect to the currently available technology, a high number of target transcriptional signature genes must be narrowed down for implementation in routine TB screening. Cepheid has been developing a novel Xpert MTB Host Response (MTB‐HR) PCR assay (not yet commercially available) based on the transcriptional signature identified by Sweeney et al. [140]. The original study described a transcriptional TB score based on differential expression of Guanylate‐Binding Protein 5 (*gbp5*), Dual Specificity Phosphatase 3 (*dusp3*), and Krüppel‐Like Factor 2 (*klf2*) genes [141]. The Xpert MTB‐HR assay detects fingerstick blood mRNA levels of these three target genes to calculate a TB score for discriminating between TB disease and other respiratory diseases [140]. Despite some promising preliminary results, a wider multicenter study recently reported an overall sensitivity of 90.3% (range of 75.9%–94.4%) and specificity of 62.6% (range of 53.4%–73.6%) compared with the microbiological reference standard, thus failing to meet the WHO minimum criteria for a triage test [142, 143]. Interestingly, the study showed 20 pp lower assay specificity with the HIV‐positive subgroup compared with the HIV‐negative subgroup (45.1% vs. 65.9%) [143]. Diagnostic approaches based on the expression of genes associated with immune function may be vulnerable to differences in distinct subpopulations such as PLHIV or children [144].

Similar approaches have recently been used to detect host response proteins as proteome signatures [145]. Compared with MDx applications, enzyme immunoassays (EIAs) are generally highly adaptable to peripheral and point‐of‐care diagnostics. Lateral flow tests represent an established application, offering minimal maintenance, rapid turnaround time, and low assay costs, although quantification of protein biomarkers may require more complex assays. Using this approach, ongoing product development has shown preliminary results meeting the WHO minimum criteria for a triage test [136]. However, this approach has similar limitations to the analysis of transcriptome signatures. Overall, emerging approaches in this field would greatly benefit from standardization and harmonization in terms of geographic location, patient demographics, and laboratory protocols to improve understanding of their feasibility in wider diagnostic use [144].

## 9. What Do We Need? Open Questions and Closing Remarks

In recent years, there has been rapid development in the field of MDxs of TB. The selection of available assays already covers the full range of the field, yet we are still dependent on conventional methods.

Many low‐resource settings still use smear microscopy instead of WHO‐recommended tests for initial diagnostics [9]. Increasing competition can partly alleviate this problem by reducing test prices, but a more affordable and simple technology is likely needed to serve near‐patient facilities, especially in low‐resource settings [146]. It has been estimated that in high‐incidence areas, increasing accessibility through peripheral point‐of‐care testing would allow a significant trade‐off in test sensitivity without reducing the overall case detection rate [147]. The recently WHO‐recommended MiniDock MTB assay is a potential solution to this need, with an estimated reduction of approximately 50% in operational costs compared with current low‐complexity NAATs, and the capability to be operated wherever needed [10, 111]. Another bold measure, stemming from the experiences in COVID‐19 testing, is the new WHO recommendation of pooling sputum samples from up to four individuals to decrease analytic costs in resource‐limited settings [10]. However, some reports suggest that the issue of accessibility to TB testing is more structural—related to a lack of healthcare infrastructure, coordination, and human resources—and thus only partially dependent on the direct costs or features of the available TB test [134, 148].

Although the WHO recommends using rapid MDx assays for initial TB testing instead of smear microscopy or culture, laboratories beyond the peripheral level performing centralized testing are still encouraged to use culture in addition to rapid diagnostics [9, 149, 150]. That is, unlike some other areas of diagnostic microbiology that may have moved toward genuine MDx screening, TB diagnostics still relies on culture. Most prominently, such a transition in TB diagnostics would require noninferior sensitivity of MDx assays compared with culture. Although diagnostic accuracy studies suggest that MDx tests can detect some cases missed by culture, meta‐analyses uniformly show that current commercial MDx tests still miss culture‐positive cases [23, 25, 27, 59, 151]. With respect to coverage of culture, some MDx tests already detect NTM, but given the distinct clinical performance rates, further development and evaluation of such assays are needed to ensure detection of the most clinically relevant cases. Beyond screening, smear microscopy and culture can also be used to monitor response to treatment [76]. Although a TB molecular bacterial load assay (TB‐MBLA) has been recently developed and shown to be more accurate than smear microscopy for treatment monitoring, this assay is currently not optimal for routine clinical diagnostics [152]. In addition to other follow‐on analyses, strain isolation by culture also allows phenotypic DST, which is especially relevant for drug‐resistant TB, as the accuracy and availability of genotypic DST for some new and repurposed drugs remain insufficient [8, 68].

In the field of rapid DST, MDx tests offer reliable results for some of the most important drugs—RIF, isoniazid (MDR‐TB), and fluoroquinolones (pre‐XDR‐TB)—but simple assays for detecting current‐definition XDR‐TB are lacking [8]. Some first‐line assays, such as the Xpert and Truenat assays, also do not include analysis for isoniazid. Especially in low‐resource settings, this limitation may significantly delay the detection of INH monoresistance, which is the most common form of TB drug resistance [12]. In general, commercial development of such tests should better keep pace with the changing drug treatment recommendations. With respect to broader‐coverage rapid DST, namely, tNGS, more research is needed to further identify the genetic factors contributing to resistance to new and repurposed drugs, as well as to develop more cost‐efficient and nonredundant approaches for implementing such assays.

Altogether, a broad selection of rapid molecular TB tests is available, covering the full spectrum of TB diagnostics and providing accurate and relevant results in days rather than several weeks. The coming years will show whether the novel, innovative testing strategies recently recommended by WHO will increase testing coverage, especially in low‐resource settings. In addition, further development of current technologies is still warranted for improving the diagnostic accuracy of MTB detection and rapid first‐ and second‐line DST. Especially in low TB endemicity settings, this progression should also cover the detection of globally increasing NTM. These steps are crucial for decreasing dependency on traditional smear microscopy and culture as routine diagnostic methods and moving toward a more comprehensive MDx approach in TB diagnostics.

## Funding

This study was funded by Tampereen Tuberkuloosisäätiö, 10.13039/501100006706, and Suomen Tuberkuloosin Vastustamisyhdistyksen Säätiö, 10.13039/501100008368

## Conflicts of Interest

The author has received a speaker fee from Cepheid.

## Data Availability

Data sharing is not applicable to this article as no datasets were generated or analyzed during the current study.
